# Adapting a Telehealth Physical Activity and Diet Intervention to a Co-Designed Website for Self-Management After Stroke: Tutorial

**DOI:** 10.2196/58419

**Published:** 2024-10-22

**Authors:** Dina Pogrebnoy, Lee Ashton, Brian A Beh, Meredith Burke, Richard Cullen, Jude Czerenkowski, Julie Davey, Amy M Dennett, Kevin English, Erin Godecke, Nicole Harper, Elizabeth Lynch, Lesley MacDonald-Wicks, Amanda Patterson, Emily Ramage, Ben Schelfhaut, Dawn B Simpson, Karly Zacharia, Coralie English

**Affiliations:** 1 School of Health Sciences University of Newcastle Newcastle Australia; 2 Department of Physiotherapy Western Health St Albans Australia; 3 Food and Nutrition Program Hunter Medical Research Institute Newcastle Australia; 4 Consumer Advisory Group Member Sydney Australia; 5 Consumer Advisory Group Member Newcastle Australia; 6 Heart and Stroke Program Hunter Medical Research Institute Newcastle Australia; 7 Stroke Foundation Melbourne Australia; 8 Consumer Advisory Group Member Wangaratta Australia; 9 Allied Health Clinical Research Office Eastern Health Melbourne Australia; 10 School of Allied Health Human Services and Sport Latrobe University Melbourne Australia; 11 Consumer Advisory Group Member Melbourne Australia; 12 Department of Speech Pathology School of Medical and Health Sciences Edith Cowan University, Sir Charles Gairdne Park Health Care Group Perth Australia; 13 Caring Futures Institute College of Nursing and Health Sciences Flinders University Adelaide Australia; 14 ASPIRE Unit Western Health Melbourne Australia; 15 Institute of Neurosciences and Mental Health Florey Melburne Australia; 16 Consumer Advisory Group Member Kent United Kingdom; 17 Centre of Research Excellence to Accelerate Stroke Trial Innovation and Translation University of Sydney Sydney Australia

**Keywords:** stroke, secondary prevention, co-design, how-to guide, website development, accessibility, navigation, self-management

## Abstract

People who experience a stroke are at a higher risk of recurrent stroke when compared with people who have not had a stroke. Addressing modifiable risk factors like physical inactivity and poor diet has been shown to improve blood pressure, a leading contributor to stroke. However, survivors of stroke often experience challenges with accessing risk reduction services including long wait lists, difficulty with transportation, fatigue, impaired function, and diminished exercise capacity. Providing health interventions via a website can extend the reach when compared with programs that are only offered face to face or via real-time telehealth. Given global challenges of accessing secondary prevention programs, it is important to consider alternative ways that this information can be made available to survivors of stroke worldwide. Using the “design thinking” framework and drawing on principles of the integrated knowledge translation approach, we adapted 2 co-designed telehealth programs called i-REBOUND – *Let’s get moving* (physical activity intervention) and i-REBOUND – *Eat for health* (diet Intervention) to create the i-REBOUND *after stroke* website. The aim of this paper is to describe the systematic process undertaken to adapt resources from the telehealth delivered i-REBOUND – *Let’s get moving* and i-REBOUND – *Eat for health* programs to a website prototype with a focus on navigation requirements and accessibility for survivors of stroke. We engaged a variety of key stakeholders with diverse skills and expertise in areas of stroke recovery, research, and digital health. We established a governance structure, formed a consumer advisory group, appointed a diverse project team, and agreed on scope of the project. Our process of adaptation had the following 3 phases: (1) understand, (2) explore, (3) materialize. Our approach considered the survivor of stroke at the center of all decisions, which helped establish guiding principles related to our prototype design. Careful and iterative engagement with survivors of stroke together with the application of design thinking principles allowed us to establish the functional requirements for our website prototype. Through user testing, we were able to confirm the technical requirements needed to build an accessible and easy-to-navigate website catering to the unique needs of survivors of stroke. We describe the process of adapting existing content and co-creating new digital content in partnership with, and featuring, people who have lived experience of stroke. In this paper, we provide a road map for the steps taken to adapt resources from 2 telehealth-delivered programs to a website format that meets specific navigation and accessibility needs of survivors of stroke.

## Introduction

### Background

A stroke happens when the blood supply to the brain is interrupted, causing oxygen deprivation and tissue death [1]. Stroke is the second leading cause of death worldwide [2]. With 1 in 4 people experiencing a stroke in their lifetime [3], it is estimated that, by the year 2030, there will be 12 million stroke-related deaths and an estimated 70 million stroke survivors globally [4]. In Australia, there are currently close to half a million people living with the effects of stroke [5], and without action, this number is predicted to exceed 800,000 by 2050 [6]. Survivors of stroke have an increased risk of stroke recurrence, with an estimated 40% of stroke survivors going on to have another stroke within 10 years [7].

Hypertension is one of the biggest risk factors for first stroke [8] and, together with physical activity and diet quality, is one of the most modifiable risk factors for recurrent stroke [8]. Adequate levels of physical activity improve blood pressure control [9], and a reduction of 10 mm Hg in systolic blood pressure has been associated with as much as a 40% reduction in stroke risk [10]. There are no trials of dietary interventions for secondary stroke prevention, but the Mediterranean-style diet holds the most promise [11]. Therefore, together with good medical management, lifestyle interventions that improve dietary behavior and physical activity levels can help reduce the risk of recurrent stroke [12,13]. Despite this, however, few programs exist to support survivors of stroke to achieve and maintain these beneficial lifestyle changes after their stroke [14].

Many survivors of stroke have difficulty with equitable access to follow-up services once discharged from hospital [15]. Common barriers include transportation [16,17], access [16], economic constraints [16], and a lack of suitable programs [16]. An additional challenge facing survivors of stroke is their diminished exercise capacity, with increased oxygen requirements during routine tasks making it hard to engage in secondary prevention programs [18]. Similarly, problems with memory or thinking as well as physical limitations, fatigue, and planning challenges can further impact a stroke survivor’s ability to participate in meaningful activity after stroke [18].

Many survivors of stroke will have lifelong effects from their stroke; therefore, resources to improve well-being after stroke need to cater for a variety of abilities and be relevant for survivors at all stages of their recovery. Unlike other chronic health conditions (eg, cardiac rehabilitation), there are no structured self-management programs beyond the initial rehabilitation phase after an acute stroke event. International clinical guidelines recommend that all survivors of stroke should receive support for self-management after their stroke [19]. A recently published Cochrane review provides recommendations on the best ways to develop effective self-management programs for survivors of stroke [19], by emphasizing that programs need to be meaningful and acceptable to people with stroke and that survivors need to be involved in program design [20].

### Previous Work

Our team developed 2 co-designed telehealth-delivered interventions to (1) increase physical activity [21] and (2) improve diet quality [22] in people after stroke. These interventions were pilot-tested in a 4-arm randomized control trial and demonstrated a clinically relevant difference in support of the interventions across a range of physical activity, diet quality, and blood pressure outcomes [23]. Building on this work, our research partners with lived experience of stroke felt that adapting our telehealth resources to be publicly available on a website was an important next step to reach more people impacted by stroke.

Websites have been used in recent years to improve accessibility to evidence-based resources for people living with complex health conditions [24,25]. Our recent review found that people living with chronic health conditions could improve their physical activity levels and aspects of diet quality if they accessed websites hosting health information [26]. Despite the apparent utility and effectiveness of websites, to the best of our knowledge, prior to the launch of the i-REBOUND *after stroke* website, there were no publicly available websites aimed at secondary stroke prevention. We hypothesized that a key barrier to creating meaningful websites for survivors of stroke is the absence of published guiding principles or functional requirements to do this well. We hoped that publishing the guiding principles used in our project will help others to develop accessible websites for survivors of stroke.

A key guiding principle of our work, as identified by our research partners with lived experience, was that the i-REBOUND *after stroke* website needed to feature survivors of stroke in the content. It was important for survivors of stroke to be able to relate to the website content, feeling that they “could only be what they could see*.*” Therefore, we created a fit-for-purpose website designed for and featuring content with survivors of stroke to address this gap.

### Objective

The purpose of this paper was to describe the systematic approach undertaken to adapt existing co-designed telehealth resources for a website prototype that meets the unique needs and priorities of people living with stroke. In this paper, we provide a road map for clinicians and researchers wanting to adapt nondigital interventions from their original form into digital-driven solutions for survivors of stroke and other similar health conditions.

## Methods

### Overview

We used the integrated knowledge translation (iKT) approach to adapt our telehealth-delivered interventions and accompanying resources into a website. The iKT approach is a coproduction approach in which “knowledge users” are included in the research team in shared partnership [27-29]. Knowledge users are described as people who administer, influence, or use the research [28,30]. Knowledge users can include health care workers, policymakers who implement research findings, and people with lived experience of the health condition of interest. This project was a partnership between the Stroke Foundation and the University of Newcastle in Australia.

### Ethical Considerations

The project commenced in April 2021 (ethical approval 2021/ETH00360 from the Hunter New England research ethics committee) and was completed in November 2022, when the i-REBOUND *after stroke* website was launched to the public. Informed consent was obtained from all research participants, and all data were deidentified prior to analysis.

### Project Plan

**Figure 1:**
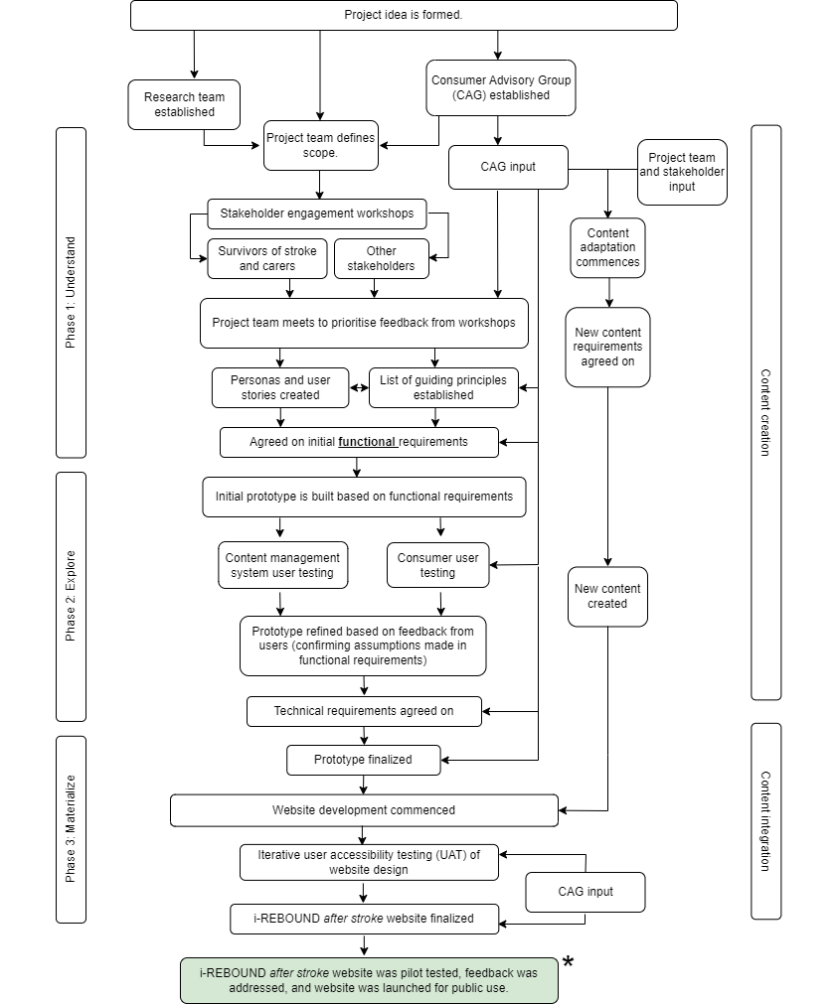
Project road map. *Findings from this phase of the project will be reported in a future paper.

Our project plan included the following 3 distinct phases, which are consistent with a design thinking framework: (1) understand, (2) explore, and (3) materialize [31]. These areas of focus are summarized in the project road map presented in Figure 1 and described in further detail in the following sections.

### Consumer Advisory Group

A Consumer Advisory Group (CAG) was convened to guide all project aspects from inception to completion. Members for the CAG were chosen to ensure representation of a range of views, experiences, and elements of lived experience. The CAG for the project included 6 people with lived experience of stroke (MB, JD, BS, BAB, KE, NH). The CAG created and agreed to a “Terms of Reference” for their engagement for the duration of this project. A copy of the “Terms of Reference” for the i-REBOUND *after stroke* website CAG can be seen in Multimedia Appendix 1. The CAG met quarterly to discuss project progress. Research leads for the project were present at the beginning of each CAG meeting and would then leave the meeting to allow for free sharing of opinions without researchers present. Cochairs of the CAG (JD and MB) were invited to all project and steering committee meetings to represent views of the CAG and to provide input from the lived experience perspective at each stage of project development. All CAG members were consulted on their preferences for ways in which they would like to offer their input to meet their communication needs.

Members of the CAG felt that the key ingredient to a successful project was to establish and maintain an authentic partnership and spirit of collaboration between members of the CAG and the broader project team. Members of the CAG identified 5 key “tips” that helped establish and maintain an authentic collaborative partnership including (1) working together from the start, (2) respecting the process as much as the outcome, (3) recognizing survivors of stroke as whole human beings, (4) being authentic and willing to learn, and (5) adopting the concept of forming an alliance with survivors of stroke. A detailed explanation of these tips can be found in Multimedia Appendix 2.

### Governance Structure

A steering committee of senior stakeholders was established to provide overall governance of the project with key deliverables being (1) progress and (2) monitoring for risks. Members included the principal investigator (CE), project lead (DP), national manager for stroke services (JC), and digital services (RC) representing the Stroke Foundation of Australia and CAG cochairs (JD and MB). The steering committee was responsible for developing and endorsing a collaboration agreement between the University of Newcastle and the Stroke Foundation and planning for sustainability of the website following public launch.

### The Project Team

Members of the project team included clinical experts in delivering physical activity and diet interventions (ER, KZ, LMW, AP, LA, AD, DP, EL, CE) as well as clinical experts in cognitive and communication difficulties (EG) for people after stroke. Other project team members included experts in co-design methodology (ER, KZ, CE), website designers (RC and LA), policymakers (JC), as well as implementation and research translation experts (EL). Most importantly, 2 research partners with lived experience of stroke (JD and MB) were members of the research team and had an equal voice at the decision-making table. The team agreed to respect everyone’s views and use a common language and strove to empower all members to share their knowledge and experience freely.

### Project Outline

#### Design Thinking Methodology

**Figure 2:**
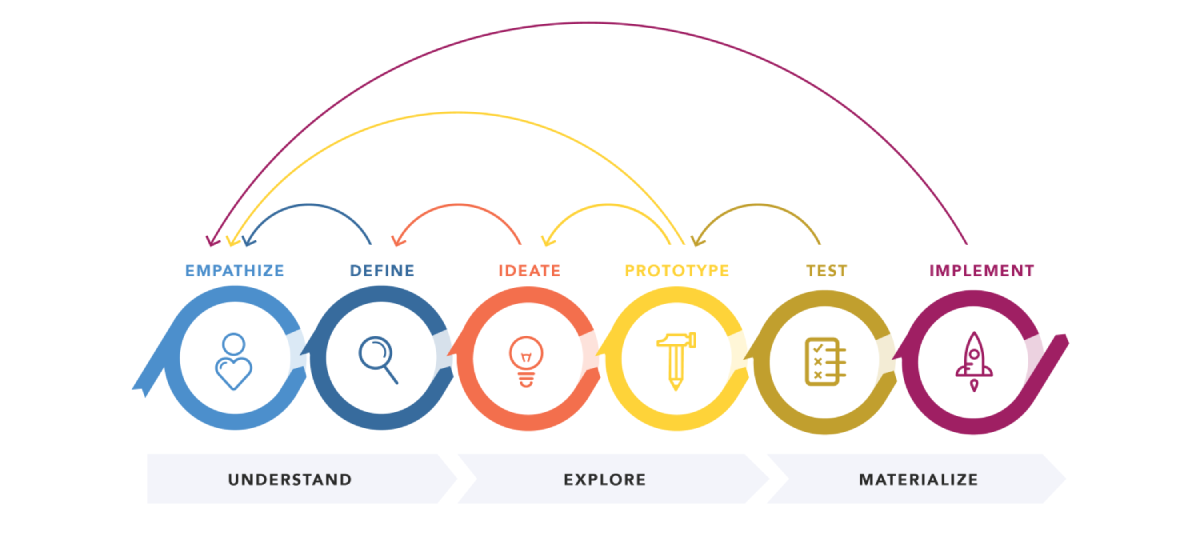
Design thinking process [31].

We used principles from the “design thinking” methodology throughout the project as outlined in Figure 2 and explained in more detail in the following sections.

Design thinking is a methodology used to tackle complex end user “problems” in a person-centric way [32]. The design thinking methodology was chosen for this project as it allowed us to identify user requirements specific to survivors of stroke.

#### Phase 1: Understand

The first key stage of phase 1, and in keeping with the iKT approach, was to engage with intended users of the i-REBOUND *after stroke* website. The “understand” phase helped to establish the functional requirements for survivors of stroke using websites with health information.

##### Stakeholder Engagement

We held workshops separately with survivors of stroke (and carers) and with other stakeholders including allied health clinicians, medical professionals, policymakers, and health care managers. We included people living with aphasia who were supported by a member of the research team who was a speech pathologist with 25 years’ experience in language disorders after stroke (EG). Workshops were facilitated by a member of the research team (DP and CE or AD) and recorded for transcribing purposes. One member of the research team facilitated the workshop with a second member of the research team there to take note of factors that might aid analysis such as passionate comments, body language, or nonverbal activity.

##### Workshops—Survivors of Stroke and Carers

The main objectives of the workshop(s) with stroke survivors and carers were to elicit perceived *barriers and facilitators* to use of websites targeting physical activity and diet quality for people with stroke and key *features* for survivors of stroke that help promote engagement and improve usability and uptake when interacting with websites.

Workshop participants were provided with an outline of the workshop (Multimedia Appendix 3) by email 1 week prior to the workshop. The outline allowed participants to formulate their thoughts on the topic and to encourage active participation in facilitated discussion at the time of the workshop. Workshops were held in small groups through an online meeting platform with breaks offered as needed. A welcome video, recorded by a CAG member (Multimedia Appendix 4) with aphasia, was played at the commencement of each workshop to encourage all participants to share their views openly. We asked participants to reflect on websites they currently use and what features help them enjoy their experience and make them want to return to the website on a future occasion. Participants were encouraged to share any difficulties they experience when using websites to help optimize the user experience of our website prototype. Participants were encouraged to comment on experiences shared by others (in relation to themselves) so that recurring themes could be captured. After the workshops, we emailed participants a written summary of discussed themes for cross-checking and invited feedback or any additional comments.

##### Workshops—“Other Stakeholders”

Stakeholder workshops were held to capture views of health professionals working in the field of stroke rehabilitation. The same process was followed with the “other stakeholders” as with survivors of stroke prior to the workshops. A copy of the workshop outline in relation to “other stakeholders” can be seen in Multimedia Appendix 5, and the welcome video can be viewed in Multimedia Appendix 6. We asked clinicians if they currently provided secondary prevention advice to their patients and in what format. Attendees discussed key considerations that they felt would help clinicians support survivors of stroke to engage with websites aimed at secondary stroke prevention. After the workshops, participants were sent a summary of discussed themes for cross-checking and were invited to provide feedback or any additional comments.

A total of 22 diverse key stakeholders (9 survivors of stroke, 1 carer, and 12 health care professionals representing dietetics, physiotherapy, speech pathology, occupational therapy, and neuropsychology, as well as medical rehabilitation specialists) contributed their views to initial workshops. A written summary of all stakeholder workshops was provided to all members of the CAG via email. The CAG met to review the content together and agree on priority focus areas to guide the project team in creating meaningful user stories and personas [33].

##### User Stories and Personas

User stories and personas are integral components of the design thinking process, aiding in the creation of more effective and user-friendly interfaces. User stories are succinct narratives describing specific user interactions with a website, structured as:

“As a *[stroke survivor]*, I want *[an action]* so that *[benefit or goal]*” [34].

User stories and user acceptance criteria for the i-REBOUND *after stroke* project were developed after stakeholder engagement workshops and are described in Multimedia Appendix 7.

Personas help website designers understand the target user and therefore humanize design decisions [35]. Personas can be useful to pick up on areas that may not have been identified in other discovery activities. Testing assumptions as part of the iterative user testing process ensured that the persona activity in our project was robust and allowed for the checking of both internal and external hypotheses. Undertaking this process ensured a higher degree of user acceptance and user-centered product design. By generating a list of meaningful personas (Multimedia Appendix 8) from the priority focus areas identified by members of the CAG, the design team could be confident that the i-REBOUND *after stroke* website would resonate with a diverse group of users [36].

##### Guiding Principles

Once we had a range of personas specific to this project, we could create a list of “guiding principles” underpinning the design and development of our website prototype. The guiding principles (Table 1) included the broad categories of design, navigation, access, function, community, language, and content display with a clear distinction between what the website design “Must be” and what it “Won’t be” from a user perspective. Members of the CAG reviewed and endorsed the list of guiding principles for the i-REBOUND *after stroke* website.

**Table 1:** Guiding principles as identified by participants in stakeholder workshops.

Guiding principle	Must be	Won’t be
General	Ability-specific Short instructions Short videos Research supported	One size fits all Long instructions Long videos Not trusted
Design	Minimal links Clean design Not a lot of steps to follow Everyday people doing everyday things	Lots of links Colorful and busy Lots of steps Unrelatable
Navigation	Clear to understand Intuitive	Confusing Complex
Access	Quick to join Accessible on multiple devices Optimized for small screens	Too long = fatigue Not optimized Not optimized
Function	Personalizable Goal-focused	Unguided Without a purpose/aim
Community	Feeling connected Encouragement	Alone Disengaged
Language	Simple Short and direct Respectful Motivational Engaging Fun Other languages	Complex Long and ambiguous Patronizing Discouraging Won’t persist Boring/dull Just English
Content displayed	Big font size Optimized for print Ad free Practical Free to use Only show information I need Easy to interpret content Sharable Stroke-related content Multimedia (images, video, and audio)	Small font Not print ready Ads or promotions Not relatable/usable Cost Oversupply of information Difficult to follow Can’t share Generic information Just text

##### Functional Requirements

Using the guiding principles, our development team was able to derive a comprehensive and user-focused list of functional requirements. Functional requirements outlined features that the i-REBOUND *after stroke* website must perform to meet the needs of our target users. These requirements specifically described what the system “should do” and how it “should behave” [37]. Our generated list of functional requirements served as the basis for designing, developing, and testing the initial i-REBOUND *after stroke* website prototype. A full list of functional requirements can be found in Multimedia Appendix 9.

#### Phase 2: Explore

##### Prototype Development

We used an iterative approach to develop the prototype to uncover design flaws, enable a process to refine features, and offer an opportunity to align the final product with user needs [38].

The initial prototype of the i-REBOUND *after stroke* website was presented to the CAG by members of the research and design team (RC, DP, CE) to seek first impressions. The CAG was then left to meet without members of the research and design team to discuss their impressions freely. The CAG provided a summary of recommendations (Multimedia Appendix 10) to the broader research team based on their review and discussion. Positive feedback was that sections were generally set up well and the “comments” section at the bottom of each page would encourage user engagement. Suggestions for improvement included creating an introduction video for each website landing page to set the scene for the purpose of each section. Another suggestion was to remove the “time I have” to cook button from each recipe and replace it with the number of steps for the recipe, as the time a recipe will take to cook is likely to vary between users with different abilities.

##### User Testing

Once the initial website prototype was ready, we proceeded to test it with a broader range of users. User testing during prototype development is a crucial step to validate design decisions and identify usability issues [39]. User testing was an important step in helping to uncover user preferences, behavior patterns, and potential pain points. To understand user experience, in relation to navigation of the i-REBOUND *after stroke* website, a member of the research team (DP) conducted 1:1 user testing sessions. User testing was completed with stroke survivors (5 members of the CAG and 2 other survivors of stroke) to help identify further development opportunities for the i-REBOUND *after stroke* website.

User testing sessions (the session guide is in Multimedia Appendix 11) were conducted using an online meeting platform and were recorded (audio and video) with consent. At the beginning of each testing session, users shared their screen and were asked to share their feelings when browsing the site, both positive and negative, to enable further analysis from the development team. During the sessions, users were asked to complete several tasks to see how easy the site was to use. If something did not feel right, users were asked to share this. Similarly, if something worked well, users were encouraged to share what was working well for them. Duration of the user testing sessions ranged from 20 minutes to 30 minutes, and breaks were offered as needed. After all user testing sessions were completed, recordings of the sessions were shared with a member of the design team. These recordings were analyzed for subtle navigation challenges that may not have been articulated through verbal feedback (eg, when it took a user an extended period to navigate somewhere but they did not say anything to indicate this was an issue).

##### Content Management System User Testing

Having a user-friendly content management system (CMS) for content managers is important for maintaining website content. CMS platforms allow users to create, edit, organize, and publish content such as text, images, videos, and documents on websites [40]. CMS user testing ensured that the content managers and administrators of the i-REBOUND *after stroke* website were able to perform key functions in the day-to-day management of the website. User testing a CMS involves evaluating the usability, functionality, and user experience of the system from the perspective of its intended user. Four members of the Stroke Foundation design and development team tested the CMS and provided feedback to website developers on areas that needed to be addressed to improve their experience.

##### Refining the Prototype

Analyzing user testing feedback, identifying technical limitations, prioritizing opportunities for enhancement, and mapping enhancements to technical requirements were important steps that needed to occur before website development could commence. Technical requirements refer to the specific capabilities, features, and resources that are necessary for a technology solution to function properly and effectively [41]. This phase of the project was completed in an iterative manner with extensive input from the CAG to agree on the minimally viable product. A minimally viable product in the context of building a website [42] needs to capture essential features and functionality to address the core needs of the target audience taking into consideration project budget and agreed timelines. CAG members guided decision-making around prioritization of enhancements as the initial prototype was refined and finalized [43]. A wish list for future enhancement opportunities was created to reflect that not all functional requirements could be addressed with the limited funding for this project.

##### Content Creation

In parallel to the process of developing the website prototype, our team worked with members of the CAG and a broader group of stroke survivors to adapt existing content from the i-REBOUND – *Let’s get moving* and i-REBOUND – *Eat for health* telehealth programs to be accessible in a digital format. An experienced physiotherapist (DP) was the lead for this phase of the project and worked closely with members of the project team to adapt existing content and create new content for the i-REBOUND *after stroke* website. The i-REBOUND *after stroke* website has 3 sections: Eat Well, Move More, and Hints and Hacks. Members of the CAG felt it was important to convey to website users that all resources on the i-REBOUND *after stroke* website were co-designed with survivors of stroke and were evidence-based to increase user confidence in the credibility of the information provided. To address this, an introductory video was created with project team members and clinical experts in eating well (LMW and KZ) and moving more (CE and ER) after stroke. A member of the CAG with lived experience of stroke (JD) introduced the Hints and Hacks section to connect with users who would most benefit from this content.

##### Content Adaptation

For the adaptation of i-REBOUND – *Eat for health* content, we used written documents created for the i- REBOUND – *Eat for health* program and adapted them to a digital format in the *Practical tips for eating well after stroke* section of the i-REBOUND *after stroke* website. Key considerations were font size and spacing, clarity of images, and keeping the language simple and consistent. Adapting existing recipes from the i-REBOUND – *Eat for health* recipe book to a digital version involved transferring all written content into templates. Reviewing the number of steps in each recipe was important to make sure all instructions were simple and aligned with feedback received in stakeholder engagement workshops. Users wanted each instruction to include an image; therefore, a key part of adapting each recipe to be accessible and user-focused was to create step-by-step images for each recipe. Images needed to be clear and appropriately reflect the written instruction.

For the adaptation of i-REBOUND – *Let’s get moving* content, we adapted the “safety instructions” and “when not to exercise” guides from the i-REBOUND – *Let’s get moving* telehealth program by making sure they were presented in an easy-to-read digital format with appropriate font size, spacing, and clear presentation. We added advice about consulting a medical professional when exercising after stroke, as users of the i-REBOUND *after stroke* website would not be supervised during exercise, which differed from the supervised telehealth programs of i-REBOUND – *Let’s get moving* and i-REBOUND – *Eat for health*. A written explanation and video about the evidence relating to recommended dose of exercise after stroke demonstrated that content on the i-REBOUND *after stroke* website was evidence-based and met clinical practice guidelines. [44]

##### New Content

The i-REBOUND *after stroke* Eat Well section contains 24 Mediterranean diet–friendly recipes as well as “Practical tips for eating well after stroke.” Members of the CAG and other stroke survivors contributed their tips about ways to simplify cooking after stroke. New content created in this section included guidance on tools when cooking one-handed, gadgets in the kitchen to conserve energy, and tips on cooking when living alone.

New content was required for the i-REBOUND *after stroke* Move More section because the exercise intervention in the telehealth program was delivered via real-time telehealth supervision by an experienced neurological physiotherapist and tailored to each participant. Our stakeholder engagement early in this project highlighted that exercise videos for the i-REBOUND *after stroke* website needed to feature stroke survivors to be relatable. In keeping with the iKT framework, we involved one of our CAG members who is a qualified personal trainer to develop a list of potential exercises (see Multimedia Appendix 12) that would be suitable for a range of abilities after stroke. Exercise choices included seated options, standing supported exercises, as well as more advanced upright options. We incorporated selections for users with an affected upper limb as well as users who used walking aids. A key criterion for each chosen exercise was for it to help the user work at a moderate level of intensity as they moved.

Members of the research team and staff at the Stroke Foundation of Australia assisted with seeking expressions of interest from survivors of stroke to help create content for the i-REBOUND *after stroke* website. We sought to represent a diverse group of stroke survivors including survivors of stroke of varied abilities, age, and the much harder to achieve aspiration of representing cultural diversity. We were able to identify 6 survivors of stroke with varied levels of functional ability and ages (39 years to 78 years old) but found it more difficult to reflect cultural diversity in our content. Therefore, people from culturally and linguistically diverse groups were underrepresented. The process of onboarding a stroke survivor to create these exercise videos had 4 stages. First, an experienced physiotherapist (DP) met with the stroke survivor (+/- carer) and discussed the key objectives of the i-REBOUND *after stroke* website and the type of content we wanted to create. Individual preferences for exercise options were discussed. Second, the physiotherapist developed a proposed exercise program and sent this for review and feedback to the stroke survivor via email. Third, a second face-to-face session was arranged in which the agreed program was practiced, adjustments were made as needed, and the proposed exercise routine was finalized. The final phase of creating the digital exercise content was the day of filming when the practiced routine was recorded at a mutually agreed location. Throughout the process of filming, stroke survivors were encouraged to share their tips for adapting exercises to suit their unique needs. The instructing physiotherapist also verbalized options to make the exercise more or less challenging to appeal to more users.

After filming, the content was edited, and a total of 34 videos showing exercise routines of different lengths (4 minutes to 35 minutes) were created. All recorded content was shared with the stroke survivor (+/- carer) for their review and approval for use prior to integrating it onto the website. Each exercise package presented on the website described the exercise routine and the functional ability for which it is suitable for in written form. Users could listen to this information (by pressing a “listen” button) if that was their preference. Each routine was presented as a full-length video with closed captions. All exercise routines on the website have a step-by-step written guide with still images describing each key component of the exercise routine. The step-by-step guide can be printed for users who prefer to have a paper guide. In addition to structured exercise routines, we filmed a variety of videos in which stroke survivors were engaged in incidental exercise in and around their home. It was important for stroke survivors to showcase these opportunities to demonstrate that any amount of moving is beneficial and there are a variety of ways to increase activity levels after stroke.

Content for the i-REBOUND *after stroke* Hints and Hacks section evolved organically as part of creating new content for the Eat Well and Move More sections. Survivors of stroke openly shared many tips that they said would have been helpful for them in the early stages of their recovery. There are 117 videos featuring a variety of tips in relation to physical activity and eating well after stroke. A key focus in this section is on what survivors of stroke “can do” rather than what they “cannot do.” This section of the website has been the most visited section and continues to receive the most positive feedback since the website was launched.

#### Phase 3: Materialize

Once the prototype was finalized, the technical requirements were handed over to the web design team to “materialize” and build the i-REBOUND *after stroke* website. This phase, like the rest of the project, relied heavily on input from people with lived experience of stroke to complete user accessibility testing (UAT) during the “build” process. This phase was important to make sure functional requirements as identified in phase 1 (understand phase) were appropriately reflected in the design of the i-REBOUND *after stroke* website. The process for UAT in this phase was the same as the process undertaken during prototype user testing in phase 2 (explore phase). Feedback from iterative UAT (2 cycles of improvement) of website navigation and accessibility by all 6 members of the CAG and 2 additional volunteers from the Stroke Foundation allowed further refinement to improve the user experience. The 2 additional volunteers from the Stroke Foundation had not been involved in prior prototype user testing to allow for a fresh perspective. Further refinement as identified through UAT included creating videos for key recommendations of the Mediterranean diet to visually demonstrate what was being recommended. We also added a specific filter for “practical tips for eating well after stroke” to the Eat Well section of the i-REBOUND *after stroke* website to make this information more accessible and easier to find. The final version of the i-REBOUND *after stroke* website was agreed on by members of the CAG and was then considered ready for pilot testing for feasibility and usability with a broader group of stroke survivors before being launched to the public.

## Discussion

### Principal Findings

The steps outlined in this paper demonstrate the systematic approach used to create the first evidence-based website co-designed with survivors of stroke targeting the reduction of risk factors associated with recurrent stroke. We appointed a multidisciplinary project team, which included the establishment of a CAG and was further enhanced by engaging a wide key stakeholder group through the life of the project. This paper provides a road map for partnering with consumers and using the design thinking methodology when adapting telehealth resources to be accessible for survivors of stroke in a website format.

Adapting existing evidence-based programs to online formats has the potential to reach more people who would otherwise not have access to this support. Incorporating stakeholder input into both website prototype design and content creation promotes sustained stakeholder engagement [45]. Several methodologies can be used when designing websites including the waterfall method [46], design thinking [32], and agile [46]. Design thinking is the preferred approach when the goal is to create an innovative solution where user experience is at the core of all decisions made [31]. By following the design thinking framework and drawing on the principles of the iKT approach, we were able to successfully adapt existing telehealth resources from the i-REBOUND – *Let’s get moving* and i-REBOUND – *Eat for health* programs to create a website that met the unique needs and priorities of people living with stroke.

Endorsement and ongoing support to promote visibility of the i-REBOUND *after stroke* website from the Australian Stroke Foundation have resulted in 89,242 page views from 22,088 unique users in over 90 countries (data from September 25, 2024) since the website was launched in November 2022 [47].

### Strengths

A strength of this work is that both the i-REBOUND – *Let’s get moving* and i-REBOUND – *Eat for health* programs were already co-designed with survivors of stroke and were effective at promoting behavior change to reduce the risk of recurrent stroke [23]. Content hosted on the i-REBOUND *after stroke* website was co-designed and evidence-based, giving users confidence in the information made available to them. Partnering with consumers throughout this project has been a key strength of this work. Taking the time to understand consumer needs through the discovery phase enabled the creation of a website prototype that met the accessibility and navigation needs of our end users. Cultivating a safe space for survivors of stroke to share freely enabled the creation of authentic and meaningful content, increasing the likelihood that the website will continue to be a well-used resource by survivors of stroke worldwide and ensuring ongoing sustainability.

Another key strength to this body of work is the partnership with Australia’s peak body responsible for initiatives to promote recovery after stroke. The i-REBOUND *after stroke* website is hosted on the main Stroke Foundation of Australia website, ensuring sustainable support for content. Links to the i-REBOUND *after stroke* website are routinely shared with newsletter subscribers of the Stroke Foundation of Australia with a clear increase to website traffic when this is done, highlighting that the content on the i-REBOUND *after stroke* website continues to be of interest to people in the stroke community. In addition to this, a member of the CAG (BAB) actively promotes the website through his weekly blogs on a variety of social media platforms, which are well followed by survivors of stroke and clinicians working in stroke recovery.

### Limitations

Although we followed a systematic approach by applying principles from the design thinking framework, there are still some limitations to the design of the i-REBOUND *after stroke* website that must be acknowledged. First, it cannot be assumed that an effective intervention via telehealth will be as effective if adapted to a self-directed website with health information. Although it appears that websites are effective for risk factor management for people with chronic illness [26], it remains unclear whether using self-directed websites with survivors of stroke will be as effective as programs like i-REBOUND – *Let’s get moving* and i-REBOUND –*Eat for health* in relation to meaningful and long-lasting behavior change [22].

Second, cost is another important limitation to producing a website that meets all user requirements and has relatable content. The relatively small budget (Aus $100,000 [US $68,610.90]) for this project meant that we had to prioritize what could be achieved in the initial phase of the project and create a wish list of future functional enhancements if further funding becomes available. Discussing these priorities with consumer partners and agreeing on a minimal viable product was an important step in making sure we delivered this project on time and on budget. Despite not being able to deliver all identified functions, we believe that the i-REBOUND *after stroke* website is the first of its kind where many key aspects of accessibility and navigation needs, as identified by people living with stroke, have been met. Equally, if additional funds are available, it would be appropriate to create content with greater representation of a culturally diverse group of stroke survivors so that content on the i-REBOUND *after stroke* website can be more relatable.

Finally, the i-REBOUND *after stroke* website, in its current state, is a static information portal where content is not customized to the user. Future enhancements to the i-REBOUND *after stroke* website should focus on the user being able to set goals in relation to being active and eating well after stroke and for website content to be customized to help them achieve these goals.

### Key Take Home Messages

Navigation and accessibility should be considered when adapting nondigital interventions to websites with information for survivors of stroke. When designing a website for survivors of stroke, members of the project team should be diverse and represent multiple levels of influence for a broad perspective. Establishing and maintaining an authentic relationship with consumer partners is critical to a successful project when utilizing principles from the design thinking methodology. The success of any project depends on how well it is set up at the beginning. Establishing a link with a peak governing body that can support the work in the long term will influence the sustainability of initial success. Engaging a wide stakeholder group early in the project enables the establishment of well-informed guiding principles that underpin future decisions. Prototyping and user-testing a website allow the confirmation of assumptions made about technical requirements from functional requirements. User-centered content, where users are not only featured in the content but are also content creators, is wanted by users who access health-related information on websites.

### Future Direction

The i-REBOUND *after stroke* website was pilot tested with stroke survivors (n=42) prior to being launched to the public. Pilot testing looked at usability via the System Usability Scale [48] and feasibility (domains of interest were “acceptability,” “demand,” and “limited efficacy”) as per the feasibility framework by Bowen et al [49]. Findings from the pilot study of the i-REBOUND *after stroke* website will be reported in a future paper. The next step in progressing this work would be to test the real-world effectiveness of the i-REBOUND *after stroke* website for patient outcomes.

### Conclusion

This paper provides a road map for the adaptation of diet and physical activity telehealth programs to an evidence-based and co-designed website with health information to promote self-management after stroke. We believe that our systematic approach, as outlined in this paper, can be used as an exemplar for any clinicians or researchers wanting to adapt any nondigital resources to a digital interface suitable to be used by survivors of stroke. Further to this, we are confident that the methods we describe in this paper could also be applied to the development of websites with health information for other consumer groups.

## Appendix

Multimedia Appendix 1 Terms of reference.

Multimedia Appendix 2 Tips for building an authentic partnership between consumer partners and researchers.

Multimedia Appendix 3 Co-design workshop outline for survivors of stroke.

Multimedia Appendix 4 Stroke survivor welcome video.

Multimedia Appendix 5 Workshop outline for other stakeholders.

Multimedia Appendix 6 Welcome video for other stakeholders.

Multimedia Appendix 7 User Stories and User Acceptability Criteria.

Multimedia Appendix 8 Personas.

Multimedia Appendix 9 Functional Requirements.

Multimedia Appendix 10 CAG feedback on prototype.

Multimedia Appendix 11 User testing guide.

Multimedia Appendix 12 Proposed exercises.

## Abbreviations

CAG: Consumer Advisory Group
CMS: content management system
iKT: integrated knowledge translation
UAT: user accessibility testing

## Data Availability

All data from this project are reported in this paper as multimedia appendices apart from interview transcripts. DP is happy to be contacted with any questions about this work.
