# Supplementary material for: Adapting a Telehealth Physical Activity and Diet Intervention to a Co-Designed Website for Self-Management After Stroke: Tutorial
Source: J Med Internet Res. 2024 Oct 22;26:e58419. doi: 10.2196/58419 (PMC11538875; doi:10.2196/58419)
Supplement: Multimedia Appendix 1 [file jmir_v26i1e58419_app1.docx]

### Appendix 1: Term of Reference for the CAG


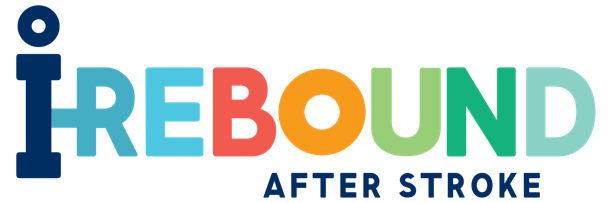


**Online**

**Consumer Advisory Group**

**Terms of Reference**

**Purpose:** To support the research project called:

“i-REBOUND *after stroke*” – development of a web-based program to prevent recurrent stroke and support long-term health and wellbeing.

The **aim** of the project is to develop a web-based resource that is designed by and features people with stroke to support being active and healthy eating to prevent secondary stroke.

**What is this about?**

The research project is led by Coralie English from the University of Newcastle.

The online program will be developed in partnership with stroke survivors in Newcastle and Melbourne.

**What will the consumer advisory group do?**

We are committed to doing what is in the best interest of people with stroke. The consumer advisory group will:

- Help us understand what is challenging and what works from the consumer perspective.
- Give advice on any changes that need to be made to the web-based platform.
- Give advice on applications for grants and other ways to get funding to expand the web-based platform.

**What is involved?**

*Stroke survivor and/or carers will be asked to:*

- Attend 3 meetings a year. The meetings will be no longer than 1 hour and will be done online (eg: zoom).
- Read some information before each meeting.
- Contribute to discussions and provide opinions.
- You might be asked to provide advice or do some work like commenting on documents in between meetings.
- The Chair(s) will be invited to attend Steering Committee meetings 2 times a year.

*Communication*

- Members of the CAG will help the wider research team understand how best to communicate between the two groups.
- A member(s) of the research team may attend the CAG meetings as an observer/facilitator if this is appropriate and requested by members of the CAG.
- If a research team member does not attend the CAG meetings, a representative from the CAG will provide a summary to the wider research team from the CAG meeting.

**How long will I be involved for?**

We would like you to be involved for a period of 2 years, until the end of 2022. This project has funding for 2 years. We may look to extend this work in the future, this will be dependent on additional funding. You will be invited to continue should you want. You can stop being involved at any time.

**What are the rules of behaviour?**

- All opinions will be listened to respectfully.
- All information discussed will be kept confidential.

**Will I be paid?**

Yes, you will be paid to attend 3 meetings per year, for 2 years. This payment includes the time spent reading information before each meeting, and for any extra work between meetings.

The amount of payment is based on the consumer council of NSW of $196.47 per meeting.
