# Supplementary material for: Adapting a Telehealth Physical Activity and Diet Intervention to a Co-Designed Website for Self-Management After Stroke: Tutorial
Source: J Med Internet Res. 2024 Oct 22;26:e58419. doi: 10.2196/58419 (PMC11538875; doi:10.2196/58419)
Supplement: Multimedia Appendix 2 [file jmir_v26i1e58419_app2.docx]

### Appendix 2: Tips from CAG members on building an authentic partnership with members of the CAG and researchers.

| Tip Title | Tip Explained |
| --- | --- |
| Work together from the start. | Each member of the CAG was invited to be actively involved in the project from the start which included setting project objectives as well as agreeing on framework and the co-design approach. |
| Respect the process as much as the outcome. | Having a good process with clear communication and set expectations led to increased involvement and better results. |
| Recognise survivors of stroke as whole human beings. | Survivors of stroke brought more to the table than ‘just’ their experience of stroke. This input added enormous value to the outcome. |
| Be real, be authentic and willing to learn. | Important to compromise and to sit with uncertainty. Ask meaningful questions and be prepared to change your thinking based on the feedback received. |
| Adopt the concept of alliance with survivors of stroke. | Survivors of stroke, just like researchers and policy makers, want to make things better for other survivors of stroke. The end goal is the same for all members of the team. |
