# Supplementary material for: Adapting a Telehealth Physical Activity and Diet Intervention to a Co-Designed Website for Self-Management After Stroke: Tutorial
Source: J Med Internet Res. 2024 Oct 22;26:e58419. doi: 10.2196/58419 (PMC11538875; doi:10.2196/58419)
Supplement: Multimedia Appendix 3 [file jmir_v26i1e58419_app3.docx]

### Appendix 3: Co-Design Workshop outline (stroke survivors)

**Co-Design Workshop outline (stroke survivors)**

**Before the workshop** can you please to consider these questions:

1. Do you **use** the **internet** to get **information** about your **health**?
2. Which **websites** do you use **regularly**?
3. Do you use **websites** for:

- **exercise**?
- **recipe ideas**?

Please give examples.

1. When you are **in a website**, what is **hard** for **you**?
2. What **features** in a website make it **easy to use**?
3. What **features**, when using a website, **help you enjoy the experience** and **want to come back** to this website?

**During the workshop**,

1. Introduction to the project and a welcome from Meredith, a stroke survivor. (approximately 5 minutes)
2. Participant introductions (approximately 5 minutes)
3. Group discussion about above questions (approximately 20 minutes)

Break (approximately 20 minutes)

1. An activity. We will ask you about our website:

| **YES - Must** have 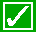 [This Photo](https://commons.wikimedia.org/wiki/File:Bright_green_checkbox-checked.svg) by Unknown Author is licensed under [CC BY-SA](https://creativecommons.org/licenses/by-sa/3.0/) | **NO – Won’t** have 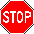 [This Photo](http://commons.wikimedia.org/wiki/File:Stop_sign_light_red.svg) by Unknown Author is licensed under [CC BY-SA](https://creativecommons.org/licenses/by-sa/3.0/) |
| --- | --- |
| **What do you want the website to be able to do?** | |

(approximately 20 minutes)

1. Summary and close. (approximately 10 minutes)

**After the workshop**, you can provide further comments if you wish.
