# Supplementary material for: Adapting a Telehealth Physical Activity and Diet Intervention to a Co-Designed Website for Self-Management After Stroke: Tutorial
Source: J Med Internet Res. 2024 Oct 22;26:e58419. doi: 10.2196/58419 (PMC11538875; doi:10.2196/58419)
Supplement: Multimedia Appendix 5 [file jmir_v26i1e58419_app5.docx]

### Appendix 5: Clinician co-design workshop - outline

**Clinician co-design workshop - outline**

**Before the workshop** can you please **consider** these questions:

1. Do you specifically provide any advice or resources around secondary stroke prevention, **diet, and physical activity in particular**?

1. Do you direct your patients to any websites for information in relation to **secondary stroke prevention**?

- If **yes**, which websites do you regularly recommend and why?
- If **no**, what stops you?

1. What do you think we need to include in our website to **engage** stroke survivors in moving more and eating well after stroke?

**Workshop** **Outline**

1. Introduction to the project and a welcome from Brian (video), a stroke survivor. (approximately 5 minutes)
2. Participant introductions (approximately 5 minutes)
3. Group discussion about above questions (approximately 30 minutes)

Break (approximately 10 minutes)

1. An activity. We will ask you about our website:

| **YES - Must** have 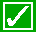 [This Photo](https://commons.wikimedia.org/wiki/File:Bright_green_checkbox-checked.svg) by Unknown Author is licensed under [CC BY-SA](https://creativecommons.org/licenses/by-sa/3.0/) | **NO – Won’t** have 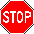 [This Photo](http://commons.wikimedia.org/wiki/File:Stop_sign_light_red.svg) by Unknown Author is licensed under [CC BY-SA](https://creativecommons.org/licenses/by-sa/3.0/) |
| --- | --- |
| **What do you think the website needs to do for you to recommend it to your patients?**  **What will make you trust it?** | |

(approximately 30 minutes)

1. Summary and close. (approximately 5 minutes)

**After the workshop**, you can provide further comments if you wish.
