# Supplementary material for: Adapting a Telehealth Physical Activity and Diet Intervention to a Co-Designed Website for Self-Management After Stroke: Tutorial
Source: J Med Internet Res. 2024 Oct 22;26:e58419. doi: 10.2196/58419 (PMC11538875; doi:10.2196/58419)
Supplement: Multimedia Appendix 7 [file jmir_v26i1e58419_app7.docx]

Appendix 7: User Stories and User Acceptance Criteria for the i-REBOUND *after stroke* website.

| **User story** | **User Acceptance criteria** |
| --- | --- |
| As a user, I want to see call to action buttons in the header navigation so I can quickly jump to key areas of the site. | # can click ‘Eat Well’ text and icon to navigate to Eat well section # can click ‘Move More’ text and icon to navigate to ‘Move More’ section # can click ‘Hints and Hacks’ text and icon to navigate to Hints and Hacks section # can click ideas text and icon to navigate to ideas section |
| As a user, I will see a Stroke Foundation global navigation on all pages, so I can quickly jump between the different Stroke Foundation websites | # can click ‘Inform Me’ to navigate to InformMe.org.au # can click ‘Enable Me’ to navigate to EnableMe.org.au # can click Stroke Foundation to navigate to strokefoundation.org.au |
| As a user, I want to see the logo in the navigation bar, so I know I am on the ‘Enable Me’/i-REBOUND website and can click back to the home page at any time. | # can click ‘Enable Me’ and i-REBOUND icon to navigate to homepage |
| As a user I can see the sign in/signup prompt to sign into I-REBOUND. | # Can see the sign in button to sign in to i-REBOUND # Can click the sign in button to be taken to a page where I can see the benefits of signing up to i-REBOUND # Can click the sign in button to be taken to a page where I can sign in # Can click the sign in button to be taken to a page where I can sign up |
| As a user, I want to see member image and dropdown to the member section, so I know I am logged in and click on it to navigate to the member options in the drop down. | # can see my image when signed in. # can see icon denoting number of items bookmarked # can click on drop down to show following options - My Items, back to ‘Enable Me’ and log out. |
| As a user, I want to see the logo in the navigation bar, so I know I am on the ‘Enable Me’/i-REBOUND website and can click back to the home page at any time. | # can click ‘Enable Me’ and i-REBOUND icon to navigate to homepage |
| As a user I can read a tag line that tells me about i-REBOUND | # Can read a brief description of i-REBOUND. |
| As a CMS user I want to be able to edit the tagline | # Can access the tagline in the content editor # Can add text and hyperlink |
| As a user I can read and click on quick link items in the footer | # Can click on About -i-REBOUND and will be taken to about i -REBOUND page, Terms of use, contact, Stroke Line. # Can click on Terms of use and will be taken to https://EnableMe.org.au/Terms-of-use # Can click on contact and will be taken to https://EnableMe.org.au/Contact # Can click on Stroke Line and will be taken to https://EnableMe.org.au/Community/Stroke Line |
| As a CMS user I want to be able to edit the link items | # Can access the link items in the content editor # Can add text and link # restrict to 4 items |
| Can see copyright and ABN info | # can read copyright and ABN text © 2021 Stroke Foundation ABN 42 006 173 379 - All donations $2 and over are tax deductible |
| As a CMS user I want to be able to edit the copyright and ABN information | # Can access the copyright info in the content editor # Can add text and link |
| Can see digital agency label | # Can read agency name # Can click on link |
| As a user I can play a video to learn about the i-REBOUND website, what it is and how to use it. | # Can click on video to watch a clip on how to use the i-REBOUND website.  # Can use a range of video tools including pause, move play bar and control sound, CC options (as expected from a YouTube embed.) |
| As a CMS user I can access the video field in the CMS and change source | #Can add a link for the video that appears |
| As a user I can read a brief intro text to understand what i-REBOUND is | # can read the title of the text # can read the body of the text |
| As a CMS user I want to edit the intro field on the homepage | # Can access the edit intro text field in the CMS (Rich text) |
| As a user I can identify the eat well collection, 3 specific items and the button to browse recipes. | # Can read title 'Eat Well after stroke' # Can click on button to browse recipes # Can see 3 items from the collection (image and title) # Can click on a recipe to go to its item page |
| As a CMS user I can select the items that appear on the home page | # Can select three items to appear on the home page # Can remove top three items so the latest items appear on home page |
| As a user I can identify the move well collection, 3 specific items and the button to browse exercises. | # Can read title 'Move More after stroke' # Can click on button to browse exercise # Can see 3 items from the collection (image and title) # Can click on an exercise to go to its item page |
| As a CMS user I can select the items that appear on the home page | # Can select three items to appear on the home page # Can remove top three items so the latest items appear on home page |
| As a user I can identify the hints and Hacks collection, 3 specific items and the button to browse hints and Hacks. | # Can read title 'Hints and Hacks' # Can click on button to browse Hints and Hacks # Can see 3 items from the collection (image and title) # Can click on a H&H to go to its item page |
| As a CMS user I can select the items that appear on the home page | # Can select three items to appear on the home page # Can remove top three items so the latest items appear on home page |
| As a user I want to see share a recipe, exercise or idea. | # Can read title that states 'Do you have a recipe, exercise or idea'  # Can click on button 'share it with us' # Can click button and taken to page with forms. |
| As a CMS user I want to edit the text in the share block | # Can edit the text of the block and link text |
| As a user I want to be interested to know more about i-REBOUND | # Can read how I can transform to a better me by using i-REBOUND |
| As a CMS user I want to edit the intro text | # Can access the intro text in the CMS and edit #Can elect to show or hide Listen/share/print buttons via checkboxes #Can select an image or video to appear on banner |
| As a user I want to read about i-REBOUND | # Can read a guide on the benefits of i-REBOUND, who is behind it, and how to incorporate it in my life to live well after stroke |
| As a CMS user I want to edit the body text | # Can access the body content area in the CMS and edit as rich text including ability to embed <> |
| As a user I want to read why I should create an account on i-REBOUND | # Can read an overview of the benefits of signing up such as bookmarking items and using member function on ‘Enable Me’. |
| As a CMS user I want to edit the Why sign-up text | # Can access the text in the CMS and edit rich text |
| As a user I want to complete the signup form to use account features of i-REBOUND | # Can see the sign-up section (duplicate ‘Enable Me’ signup as account is same) #if my email is already part of enable me registered users, I want to be told to sign in with my ‘Enable Me’ account |
| As a user I want to sign in to i-REBOUND so I can use the ability to bookmark items | # Can see the sign in section # Can enter email address # Can enter password # Can show password # Can click on forgot your password to reset # Can click sign in button |
| As a user I can be lured to share my Hints and Hacks, recipes, or exercises | # Can read an overview of What is sharing, why I should share and how to share. |
| As a CMS user I want to edit the intro text | # Can access the intro text in the CMS and edit |
| As a user I can chose one of 3 forms to share my own ideas for a recipe, exercise or hint and tip | # Can see three options for each section, recipes, exercise and Hints and Hacks. # can select an option upon which a form appears  #can see each form is custom to each section. |
| As a CMS user I want to change the embeds if forms change. | # Can access and edit the form embed for each form |
| As a user I can play a video to learn about eating well after stroke | # can click on video to watch a clip-on healthy eating.  #can use a range of video tools including pause, move play bar and control sound, CC options (as expected from a YouTube embed.) |
| As a CMS user I can access the video field in the CMS and change source | # Can add a link for the video that appears # Can add image rather than video in block |
| As a user I can read a brief intro text to understand the eat well after stroke section | # can read the title of 'Eat Well after stroke' # can read the body of the text underneath |
| As a CMS user I want to edit the intro text | # Can access the intro text in the CMS and edit Rich text |
| As a user I want to select a filter item so I can narrow in on the items I am interested in | # Can select one or multiple filter items to narrow my search # Can see the box outline change colour as I hover an item # Can see the items I have selected by the way they change colour (inverted) # Will launch with the items [breakfast][lunch][dinner][sides][snacks][salads][quick & easy][vegetarian][vegan][gluten free][dairy free][swallowing] # Can clear filters so I can start filtering again # Can select more filters if there are more than six in the filter section |
| As a user I want to browse the collection of recipes so I can identify the recipe I would like to make. | # Can see a 4x3 grid of recipes # Can see recipe title # Can see recipe image # Can see the number of steps # Can select load more to see more recipes # Can see number of recipes found (needs design) |
| As a CMS user I want to be able to prioritise items so they appear high up in the filter | # Can access an item and give it priority to appear higher up in the collection |
| As a CMS user I want to be able to apply multiple tags so filters will work | # Can tag out of breakfast, lunch, dinner, sides, snacks, salads, quick & easy, vegetarian, vegan, gluten free, dairy free, swallowing |
| As a user I can play a video to learn about moving more after stroke | # can click on video to watch a clip on moving more.  #can use a range of video tools including pause, move play bar and control sound, CC options (as expected from a YouTube embed.) |
| As a CMS user I can access the video field in the CMS and change source | # Can add a link for the video that appears # Can add image rather than video in block |
| As a user I can read a brief intro text to understand the moving well after stroke section | # can read the title of 'Move More after stroke' # can read the body of the text underneath |
| As a CMS user I want to edit the intro text | # Can access the intro text in the CMS and edit Rich text |
| As a user I want to select a filter item so I can narrow in on the items I am interested in | # Can select one or multiple filter items to narrow my search # Can see the box outline change colour as I hover over an item # Can see the items I have selected by the way they change colour (inverted)  # Can clear filters so I can start filtering again # Can select more filters if there are more than six in the filter section |
| As a user I want to browse the collection of exercises so I can identify the exercise I would like to do. | # Can see a 4x3 grid of exercises # Can see exercise title # Can see exercise image  # Can select load more to see more exercises # Can see number of exercises found (needs design) |
| As a CMS user I want to be able to prioritise items so they appear high up in the filter | # Can access an item and give it priority to appear higher up in the collection |
| As a CMS user I can apply multiple tags to an exercise so that filters will work | # Can access an item and add multiple tags. (1 minimum) |
| As a user I can play a video to learn about Hints and Hacks after stroke | # Can click on video to watch a clip on Hints and Hacks.  # Can use a range of video tools including pause, move play bar and control sound, CC options (as expected from a YouTube embed.) |
| As a CMS user I can access the video field in the CMS and change source | # Can add a link for the video that appears # Can add image rather than video in block |
| As a user I can read a brief intro text to understand the hints and Hacks section | # Can read the title of 'Hints & Hacks to live well after stroke' # Can read the body of the text underneath |
| As a CMS user I want to edit the intro text | # Can access the intro text in the CMS and edit Rich text |
| As a user I want to select a filter item so I can narrow in on the items I am interested in | # Can select one or multiple filter items to narrow my search # Can see the box outline change colour as I hover an item # Can see the items I have selected by the way they change colour (inverted) # Will launch with the items [eating well][moving well] # Can clear filters so I can start filtering again # Can select more filters (that dropdown) if there are more than six in the filter section |
| As a user I want to browse the collection of Hints and Hacks so I can identify the hint and hack I would like to read. | # Can see a 4x3 grid of recipes # Can see recipe title # Can see recipe image # Can see number of steps # Can select load more to see more recipes # Can see number of recipes found (needs design) |
| As a CMS user I want to be able to prioritise items so they appear high up in the filter | # Can access an item and give it priority to appear higher up in the collection |
| As a CMS user I can apply multiple tags so filters will work | # Can access an item and add multiple tags. (1 minimum) |
| As a user I want to read about the profile section on i-REBOUND and how to use it | # Can read my saved bookmarks section to know what the page is # Can see button to create a goal (take me to the ‘Enable Me’ goal setting section) # Can see button to download exercise tracking sheet (pdf) |
| As a user I want to watch a video on my profile section so I know how to use it | # Can watch a video (embedded YouTube) # Can view an image |
| As a user I want filter my bookmarks | # Can read filter title  # Can filter items between [Eat Well] [Move More][Hints and Hacks] |
| As a user I want to view my bookmarks, click on one to view it, or remove an item from my bookmarks | # Can see all items bookmarked # Can see number of items bookmarked # Can select item to read more # Can choose to remove an item from my collection |
| As a user I want to read an Item title and brief description, see tagged items and then have the option to save, print, share or listen to item | # Can read the title of the item # Can read a brief description of the item # Can go back to collection by clicking on back to hints and Hacks # Can save item to my bookmarks # Can see when i save item that it has moved to my bookmarks # Can print a print friendly version of the page # Can share item on social media (use existing 'add this' as on ‘Enable Me’) # Can play read-speaker button to hear content read aloud # Can see icons for tagged all tagged items |
| As a CMS user I can add title, description and icons and text so I can add an item to the eat well collection | # Can add title # Can add description # Can add icons and text # Can add my own button, text and link  * Control over ‘other’ button not required |
| As a user I want to watch a video or view an image of the dish I am interested in | # Can watch a video (embedded YouTube) # Can view an image |
| As a CMS user I can choose to add a video or image | # Can add video link into CMS # Can add image instead of video |
| As a user I can see what I need to undertake a recipe | # Can see the title 'What you need' # Can see for how many people these ingredients will make for # Can see individual items (text) I need to undertake a recipe |
| As a CMS user I can add items that you need to complete the activity | # Can add item image # Can add item text # Can add link in the text # Can choose not to have image |
| As a user I can see all the steps to complete a recipe so I can follow along | # Can see the title 'What to do' # Can see step by step instructions (Step#, text and image) # Can see link if required |
| As a CMS user I want to add step by step instructions with numbering. | # Can add multiple steps # Can add text with links in step instruction # Can add sub text item below instruction with text link # Can add image or video # Can click on image and it expands # Can click on video and it opens in lightbox. |
| As a user I want to comment on a recipe | # Can add a comment to a recipe. Cannot add links, images or videos. # Can scroll down page to read more comments (no pagination) |
| As a user I want to read an Item title and brief description, see tagged items and then have the option to save, print, share, listen to item, or download tracking sheet | # Can read the title of the item # Can read a brief description of the item # Can go back to collection by clicking on back to Hints and Hacks # Can save item to my bookmarks # Can see when I save item that it has moved to my bookmarks # Can print a print friendly version of the page # Can share item on social media (use existing 'add this' as on ‘Enable Me’) # Can play read-speaker button to hear content read aloud # Can download exercise tracking sheet |
| As a CMS user I can add title, description and icons and text so I can add an item to the move well collection | # Can add title # Can add description # Can add icons and text # Can add my own button, text and link  * Control over other buttons (print share, listen) not required |
| As a user I want to watch a video or view an image of the exercise I’m interested in | # Can watch a video (embedded YouTube) # Can view an image |
| As a CMS user I can choose to add a video or image | # Can add video link into CMS # Can add image instead of video |
| As a user I can see what I need to undertake an exercise | # Can see the title 'What you need' # Can see individual items (text and icon) i need to undertake an exercise |
| As a CMS user I can add items that you need to complete the activity | # Can add item image # Can add item text # Can add link in the text # Can choose not to have image |
| As a user I can see all the steps to complete an exercise so I can follow along | # Can see the title 'What to do' # Can see step by step instructions (step#, text and image) # Can see link if required |
| As a CMS user I want add step by step instructions with numbering. | # Can add multiple steps # Can add text with links in step instruction # Can add sub text item below instruction with text link # Can add image or video # Can click on image and it expands # Can click on video and it opens in lightbox. |
| As a user I want to comment on a recipe | # Can add a comment to a recipe. Cannot add links, images or videos. # Can scroll down page to read more comments (no pagination) |
| As a user I want to read an Item title and brief description and then have the option to save, print or share item | # Can read the title of the item # Can read a brief description of the item # Can go back to collection by clicking on back to h]Hints and Hacks # Can save item to my bookmarks # Can see when I save item that it has moved to my bookmarks # Can print a print friendly version of the page # Can share item on social media (use existing 'add this' as on ‘Enable Me’) # Can play read-speaker button to hear content read aloud |
| As a CMS user I can add title, description and icons and text so I can add an item to the move well collection | # Can add title # Can add description # Can add icons and text # Can add my own button, text and link  * Control over other buttons (print share, listen) not required |
| As a user I want to watch a video or view an image of the item I’m interested in | # Can watch a video (embedded YouTube) # Can view an image |
| As a CMS user I can choose to add a video or image | # Can add video link into CMS # Can add image instead of video |
| As a user I want to read about a hint and tip | # Can read the main content of hint and tip |
| As a CMS user I want to add content | Rich text |
| As a user I want to comment on a recipe | # Can add a comment to a recipe. Cannot add links, images or videos. # Can scroll down page to read more comments (no pagination) |
| As a user I can play read-speaker tool so I can hear back the audio of written text on a page | # Can play read speaker on pages to play back page content |
| As a user I want to easily use this site on mobile and tablet | # Can view the site on mobile device. # Can see the site is optimised for tablet. |
| As a user I can complete a survey to provide the i-REBOUND team with behaviour change measures of program | # Can answer how often I use i-REBOUND # Can answer what is my measure of physical activity # Can answer what is my measure of dietary intake # Can answer question regarding my self-efficacy in using the site # Can answer questions regarding my readiness to change # Can answer overall effectiveness of site # Can answer if I would recommend this site to others # Can see call to action to set a goal # Can be contacted for user testing in the future |
| As a user I can complete a survey to provide the i-REBOUND team with behaviour change measures of program | # Can answer how often I use i-REBOUND # Can answer what is my measure of physical activity # Can answer what is my measure of dietary intake # Can answer question regarding my self-efficacy in using the site # Can answer questions regarding my readiness to change # Can answer overall effectiveness of site # Can answer if I would recommend this site to others # Can see call to action to set a goal # Can be contacted for user testing in the future |
| As a user I can complete a survey to provide the i-REBOUND team with behaviour change measures of program | # Can answer how often I use i-REBOUND # Can answer what is my measure of physical activity # Can answer what is my measure of dietary intake # Can answer question regarding my self-efficacy in using the site # Can answer questions regarding my readiness to change # Can answer overall effectiveness of site # Can answer if I would recommend this site to others # Can see call to action to set a goal # Can be contacted for user testing in the future |
| As a user I can complete a survey to provide the i-REBOUND team with behaviour change measures of program | # Can answer how often I use i-REBOUND # Can answer what is my measure of physical activity # Can answer what is my measure of dietary intake # Can answer question regarding my self-efficacy in using the site # Can answer questions regarding my readiness to change # Can answer overall effectiveness of site # Can answer if I would recommend this site to others # Can see call to action to set a goal # Can be contacted for user testing in the future |
| As a user I can complete a survey to provide the i-REBOUND team with evaluation of program | # Can answer on overall effectiveness of platform # Can answer on profile, current physical activity, risk factors, readiness to change |
| As a user I can complete a survey to provide the i-REBOUND team with evaluation of program | # Can answer on overall effectiveness of platform # Can answer on profile, current physical activity, risk factors, readiness to change |
