# Supplementary material for: Adapting a Telehealth Physical Activity and Diet Intervention to a Co-Designed Website for Self-Management After Stroke: Tutorial
Source: J Med Internet Res. 2024 Oct 22;26:e58419. doi: 10.2196/58419 (PMC11538875; doi:10.2196/58419)
Supplement: Multimedia Appendix 8 [file jmir_v26i1e58419_app8.docx]

Appendix 8: List of personas from priority focus areas identified by members of the CAG as part of the i-REBOUND *after stroke* project.

| **Health Information Source** | **Barriers** | **Condition/Challenge** | **Guiding principle** |
| --- | --- | --- | --- |
| Adam seeks information from people he trusts and who have a say in his care. He relies on information from his GP and friends. He had to adopt a lot of changes to his lifestyle after his stroke and is hesitant to learn too many new things outside his comfort zone. | not aware of information out there. Feels he has made a lot of changes and that is ok. Worried that exerting himself into new things has both physical and mental fatigue impacts. | Aphasia, Fatigue | Simple > complex  push > pull personal > unguided Quick > long intuitive > mentally process cues |
| Bai relies on her Husband to find information on stroke and relay it to her. He often prints her out sections of sites or forums or shows her useful videos. She struggles to independently use computers. | Bai can often feel overwhelmed by her stroke. The amount of information, and the effort to understand medical terms has made her reluctant to focus on anything other than managing her fatigue and wanting to cook again. | Mild leg function Arm function Fatigue | Printable/emailable>so i don’t have to go online easy English> English is not first language Goal focused> not purposeful Dependent>independent need to see improvement>no progress demotivation |
| Carla searches for information to better explain her condition. She hears terms that she doesn't understand and looks online to better comprehend at her own pace and find information that relates to her. She also likes to read books on health but has struggled since her stroke. Struggle to types since her stroke but uses voice search. | Since her stroke Carla wants to know how she can prevent this from happening again and how to improve her aphasia and physical movement. She has found she needs visual cues and is more comfortable connecting with other online rather than in person. | Aphasia leg movement | Easy English > Medical terms positivity>realism Steps forward>static encourage>realism learn from others> learn by myself Why me? how can i stop this from happening again> |
| Daniel searches via google to receive lots of information and determine most relevant. He likes to have a rounded understanding of topics and know the why. He feels he makes better decisions/ actions by having an open mind and understanding topics in this way. He likes videos that show how to do things in better ways (life hacks). | Likes clarity and directness with information and instructions. Struggle with taking on too much information. Can only watch video 3-5m long. With all activities he makes very conscious decisions on how much he can give to it to avoid fatigue. | Fatigue/Speech Memory |  |
| Trust and credibility are very important to Edith. She looks for official websites rather than searching via google. She has strong opinions on what resources are important. She likes connecting with others and sharing. She believes it is important to set goals in her recovery and a lot of the information she looks for reflects her goals. | She would like to have a person who could motivate her. A buddy who she could chat to and share stories with. This would help motivate her. | Aphasia, Cognitive, Fatigue | Quick information > browsing multimedia > all text Connection > left alone motivation > left alone bookmark>remember option to show more information>showing all information positive messaging>balanced community endorsement>self-discovery Community>individual |
| Frank spends a lot of time online and conducts his own research. He resonates with sites that have been codesigned and constructed with its users in mind and information that is personal and created by those who have experienced it. He watches a lot of videos on health and shares it with his family and friends. | Frank is very self-motivated and this has assisted him greatly in his recovery but he has what he would consider invisible barriers. Hi memory is greatly impacted and he needs to write a lot down in note pad. His resilience has also come at a cost in terms of his fatigue and he struggle to maintain his energy and expenditure. | Memory Mild Aphasia Fatigue | Guided > custom remembers me > forgets remembers my progress> forgets Positive>non emotive Motivation>balance/realism Relatable>distant Connection>isolated Learning for others>isolated |
| Is a member of young stroke support groups and feel well connected to what is going on. Sees her stroke as a turning point to change her life for the better and spends a lot of time looking at health information for mind and body. She lives on her phone and uses this to connect with others and information. |  |  |  |
| Would like to spend more time researching what is available but given her load as carer finds it difficult. Would like to be better connected to stroke carers so she doesn’t have to look around and can also have support of others. Has found it difficult to find this support. | Finds it difficult to find the right exercise programs and motivation. Feels given her partners physical limitations there needs to be programs more directed at low ability. Would like to encourage her partner to be self-motivated as she worries without her it wouldn’t happen. | Financial. Partner has fatigue and struggles with basic activities and exercise due to leg function impairment. Finds it very difficult to motivate her partner. | Motivated> Positive> Start simple> |
| Finds it hard to find the right information. Would like to have more help for carers and be connected with information relevant to carers. Not so much interested in chatting to other carers. Wants to be connected to right information/new information as quickly as possible. |  | Family |  |
| Often sends people to the Stroke Foundation site but often hears that there are too many options available and that people get lost. It is too overwhelming. Jane talks to patients in rehab about secondary stroke prevention during admission. We always revisit this | education. timing of information/overwhelming people are more receptive further down the track. Believes that it is important for stroke survivors to learn from others when learning about information and to provide some inspiration and motivation. | Time. Would like to see strategies in place for survivors to manage their fatigue. Often feels that such interactions do not warn or provide this and can often fail. | Accountability>left alone Gamification>no engagement, motivation Realistic> unrealistic practical>of no use/hard to connect with Reminders>forget |
| Karim will offer the iPad on ward for site such as Enable Me. Provides information/booklets on secondary risk prevention. | Found people who used the internet before their stroke found it a lot easier. Found certain age groups or demographics were not interested in online information and it needed to be printed. |  |  |
| Refers to Enable Me and Stroke Foundation site and their factsheets. Own program pivoted to online/telehealth after Covid. Nutrition department has their own resources that she directs them too as well. Will be customed to the resources she feels they need. Does follow up to see how they have understood it. | Will often show videos from trusted to websites to provide information in different and engaging ways. Wants survivors to feel trust with websites and that they can be easy to use and have the information they need. Great deal of effort is in confidence building and showing information that has practical use for the person. | Changing model from in person programs to telehealth during Covid. Has found it is important to ask patient what they have just learnt. Worries that online experience may not reinforce important messages. Would like to see interaction and engagement. |  |
