# Supplementary material for: Adapting a Telehealth Physical Activity and Diet Intervention to a Co-Designed Website for Self-Management After Stroke: Tutorial
Source: J Med Internet Res. 2024 Oct 22;26:e58419. doi: 10.2196/58419 (PMC11538875; doi:10.2196/58419)
Supplement: Multimedia Appendix 9 [file jmir_v26i1e58419_app9.docx]

### Appendix 9: List of Functional requirements

| **Website Feature** | **User story** | **User Acceptance criteria/Functional requirement** |
| --- | --- | --- |
| **Header** | | |
| **Call to action buttons** | As a user, I want to see call to action buttons in the header navigation so I can quickly jump to key areas of the site. | # can click Eat well text and icon to navigate to Eat well section # can click Move well text and icon to navigate to Move well section # can click Hints and Tips text and icon to navigate to Hints and Tips section # can click ideas text and icon to navigate to ideas section |
| **Stroke Foundation website global navigator** | As a user, I will see a Stroke Foundation global nav on all pages, so I can quickly jump between the different Stroke Foundation websites | # can click informme to navigate to informme.org.au # can click enableme to navigate to enableme.org.au # can click stroke foundation to navigate to strokefoundation.org.au |
| **Logo** | As a user, I want to see the logo in the navigation bar, so I know I am on the enableme/i-rebound website and can click back to the home page at any time. | # can click enableme and i-REBOUND icon to navigate to homepage |
| **Member logged out** | As a user I can see the sign in/signup prompt to sign into I-rebound. | # Can see the sign in button to sign in to i-rebound # Can click the sign in button to be taken to a page where i can see the benefits of signing up to i rebound # Can click the sign in button to be taken to a page where i can sign in # Can click the sign in button to be taken to a page where i can sign up |
| **Member logged in** | As a user, I want to see member image and dropdown to the member section, so I know I am logged in and click on it to navigate to the member options in the drop down. | # can see my image when signed in. # can see icon denoting number of items bookmarked # can click on drop down to show following options - My Items, back to enableme, and log out. |
| **Footer** | | |
| **Logo** | As a user, I want to see the logo in the navigation bar, so I know I am on the enableme/i-REBOUND website and can click back to the home page at any time. | # can click enableme and i-rebound icon to navigate to homepage |
| **Tagline** | As a user i can read a tag line that tells me about i-rebound | # Can read a brief description of irebound. |
| **Tagline (CMS)** | As a CMS user i want to be able to edit the tagline | # Can access the tagline in the content editor # Can add text and hyperlink |
| **Footer link items** | As a user i can read and click on quick link items in the footer | # Can click on About -irebound and will be taken to about i rebound page, Terms of use, contact, strokeline. # Can click on Terms of use and will be taken to https://enableme.org.au/Terms-of-use # Can click on contact and will be taken to https://enableme.org.au/Contact # Can click on strokeline and will be taken to https://enableme.org.au/Community/Strokeline |
| **Footer link items (CMS)** | As a CMS user i want to be able to edit the link items | # Can access the link items in the content editor # Can add text and link # restrict to 4 items |
| **Copyright / ABN** | Can see copyright and abn info | # can read copyright and abn text © 2021 Stroke Foundation ABN 42 006 173 379 - All donations $2 and over are tax deductible |
| **Copyright / ABN (CMS)** | As a CMS user i want to be able to edit the copyright and ABN information | # Can access the copyright info in the content editor # Can add text and link |
| **Digital agency label** | Can see digital agency label | # Can read agency name # Can click on link |
| **Homepage** | | |
| **Video** | As a user i can play a video to learn about the i-rebound, what it is and how to use it. | # Can click on video to watch a clip on how to use i-rebound.  # Can use a range of video tools including pause, move play bar and control sound, Closed Caption options (as expected from a YouTube embed.) |
| **Video (CMS)** | As a CMS user i can access the video field in the CMS and change source | #Can add a link for the video that appears |
| **Intro text** | As a user i can read a brief intro text to understand what i-rebound is | # can read the title of the text # can read the body of the text |
| **Intro text (CMS)** | As a CMS user i want to edit the intro ext field on the homepage | # Can access the edit intro text field in the CMS (Rich text) |
| **Eat well collection quick block** | As a user i can identify the eat well collection, 3 specific items and the button to browse recipes. | # Can read title 'Eat well after stroke' # Can click on button to browse recipes # Can see 3 items from the collection (image and title) # Can click on a recipe to go to its item page |
| **Eat well collection quick block (CMS)** | As a CMS user i can select the items that appear on the home page | # Can select three items to appear on the home page # Can Remove top three items so the latest items appear on home page |
| **Move well collection quick block** | As a user i can identify the move well collection, 3 specific items and the button to browse exercises. | # Can read title 'Move well after stroke' # Can click on button to browse exercise # Can see 3 items from the collection (image and title) # Can click on an exercise to go to its item page |
| **Move well collection quick block (CMS)** | As a CMS user i can select the items that appear on the home page | # Can select three items to appear on the home page # Can Remove top three items so the latest items appear on home page |
| **Hint and tips collection quick block** | As a user i can identify the hints and tips collection, 3 specific items and the button to browse hints and tips. | # Can read title 'Hints and tips' # Can click on button to browse hints and tips # Can see 3 items from the collection (image and title) # Can click on a H&T to go to its item page |
| **Hint and tips collection quick block (CMS)** | As a CMS user i can select the items that appear on the home page | # Can select three items to appear on the home page # Can Remove top three items so the latest items appear on home page |
| **Share block** | As a user i want to see share a recipe, exercise or idea. | # Can read title that states 'Do you have a recipe, exercise or idea'  # Can click on button 'share it with us' # Can click button and taken to page with forms. |
| **Share block (CMS)** | As a CMS user i want to edit the text in the share block | # Can edit the text of the block and link text |
| **About i-REBOUND page (generic page template)** | | |
| **Intro text** | As a user i want to be interested to know more about i-REBOUND | # Can read how i can transform to a better me by using i-REBOUND |
| **Intro text (CMS)** | As a CMS user i want to edit the intro text | # Can access the intro text in the CMS and edit #Can elect to show or hide Listen/share/print buttons via checkboxes #Can select an image or video to appear on banner |
| **Body text** | As a user i want to read about i rebound | # Can read a guide on the benefits of i-REBOUND, who is behind it, and how to incorporate it in my life to live well after stroke |
| **Body text (CMS)** | As a CMS user i want to edit the body text | # Can access the body content area in the CMS and edit as rich text including ability to embed <> |
| **Sign up page.** | | |
| **Why sign up** | As a user i want to read why i should create an account on i-REBOUND | # Can read an overview of the benefits of signing up such as bookmarking items and using member function on enableme. |
| **Why sign up (CMS)** | As a CMS user i want to edit the Why sign-up text | # Can access the text in the CMS and edit rich text |
| **Sign up section** | As a user i want to complete the signup form to use account features of i-rebound | # Can see the sign-up section (duplicate enableme signup as account is same) #if my email is already part of enable me registered users, i want to be told to sign in with my enableme acct |
| **Sign in section** | As a user i want to sign in to i-rebound so i can use the ability to bookmark items | # Can see the sign in section # Can enter email address # Can enter password # Can show password # Can click on forgot your password to reset # Can click sign in button |
| **Share form page (generic page template)** | | |
| **Share intro text** | As a user i can be lured to share my tips, recipe, or exercise | # Can read an overview of What is sharing, why i should share and how to share. |
| **Share intro text (CMS)** | As a CMS user i want to edit the intro text | # Can access the intro text in the CMS and edit |
| **Eat well Landing Page** | | |
| **Video** | As a user I can play a video to learn about eating well after stroke | # can click on video to watch a clip-on healthy eating.  #can use a range of video tools including pause, move play bar and control sound, CC options (as expected from a YouTube embed.) |
| **Video (CMS)** | As a CMS user i can access the video field in the CMS and change source | # Can add a link for the video that appears # Can add image rather than video in block |
| **Intro text** | As a user i can read a brief intro text to understand the eat well after stroke section | # can read the title of 'Eat well after stroke' # can read the body of the text underneath |
| **Intro text (CMS)** | As a CMS user i want to edit the intro text | # Can access the intro text in the CMS and edit Rich text |
| **Filter** | As a user i want to select a filter item so i can narrow in on the items i am interested in | # Can select one or multiple filter items to narrow my search # Can see the box outline change colour as i hover an item # Can see the items i have selected by the way they change colour (inverted) # Will launch with the items [breakfast][lunch][dinner][sides][snacks][salads][quick & easy][vegetarian][vegan][gluten free][dairy free][swallowing] # Can clear filters so i can start filtering again # Can select more filters if there are more than six in the filter section |
| **Recipe collection** | As a user i want to browse the collection of recipes so i can identify the recipe i would like to make. | # Can see a 4x3 grid of recipes # Can see recipe title # Can see recipe image # Can see number of steps # Can select load more to see more recipes # Can see number of recipes found (needs design) |
| **Recipe collection (CMS)** | As a CMS user i want to be able to prioritise items so they appear high up in the filter | # Can access an item and give it priority to appear higher up in the collection |
| **Recipe collection (CMS)** | As a CMS user I want to be able to apply multiple tags so filters will work | # Can tag out of breakfast, lunch, dinner, sides, snacks, salads, quick & easy, vegetarian, vegan, gluten free, dairy free, swallowing |
| **Move well Landing Page** | | |
| **Video** | As a user i can play a video to learn about moving well after stroke | # can click on video to watch a clip on moving well.  #can use a range of video tools including pause, move play bar and control sound, CC options (as expected from a YouTube embed.) |
| **Video (CMS)** | As a CMS user i can access the video field in the CMS and change source | # Can add a link for the video that appears # Can add image rather than video in block |
| **Intro text** | As a user i can read a brief intro text to understand the moving well after stroke section | # can read the title of 'Move well after stroke' # can read the body of the text underneath |
| **Intro text (CMS)** | As a CMS user i want to edit the intro text | # Can access the intro text in the CMS and edit Rich text |
| **Filter** | As a user i want to select a filter item so i can narrow in on the items i am interested in | # Can select one or multiple filter items to narrow my search # Can see the box outline change colour as i hover an item # Can see the items i have selected by the way they change colour (inverted) # Will launch with the items [xxxxxxxx][xxxxxxxx] # Can clear filters so I can start filtering again # Can select more filters if there are more than six in the filter section |
| **exercise collection** | As a user I want to browse the collection of exercises so I can identify the exercise I would like to do. | # Can see a 4x3 grid of exercises # Can see exercise title # Can see exercise image # Can see amount of [xxxxxxx] # Can select load more to see more exercises # Can see amount of exercises found (needs design) |
| **exercise collection (CMS)** | As a CMS user i want to be able to prioritise items so they appear high up in the filter | # Can access an item and give it priority to appear higher up in the collection |
| **exercise collection (CMS)** | As a CMS user I can apply multiple tags to an exercise so that filters will work | # Can access an item and add multiple tags. (1 minimum) |
| **Hints & Tips Landing Page** | | |
| **Video** | As a user i can play a video to learn about hints and tips after stroke | # Can click on video to watch a clip on hints and tips.  # Can use a range of video tools including pause, move play bar and control sound, CC options (as expected from a YouTube embed.) |
| **Video (CMS)** | As a cms user i can access the video field in the CMS and change source | # Can add a link for the video that appears # Can add image rather than video in block |
| **Intro text** | As a user i can read a brief intro text to understand the hints and tips section | # Can read the title of 'Hints & tips to live well after stroke' # Can read the body of the text underneath |
| **Intro text (CMS)** | As a CMS user i want to edit the intro text | # Can access the intro text in the CMS and edit Rich text |
| **Filter** | As a user i want to select a filter item so i can narrow in on the items i am interested in | # Can select one or multiple filter items to narrow my search # Can see the box outline change colour as i hover an item # Can see the items i have selected by the way they change colour (inverted) # Will launch with the items [eating well][moving well] # Can clear filters so i can start filtering again # Can select more filters (that dropdown) if there are more than six in the filter section |
| **Hints and Tips collection** | As a user i want to browse the collection of hints and tips so i can identify the hint and tip i would like to read. | # Can see a 4x3 grid of recipes # Can see recipe title # Can see recipe image # Can see amount of steps # Can select load more to see more recipes # Can see amount of recipes found (needs design) |
| **Hints and Tips collection (CMS)** | As a CMS user i want to be able to prioritise items so they appear high up in the filter | # Can access an item and give it priority to appear higher up in the collection |
| **Hints and Tips collection (CMS)** | As a CMS user I can apply multiple tags so filters will work | # Can access an item and add multiple tags. (1 minimum) |
| **My Profile** | | |
| **Saved bookmarks intro block** | As a user i want to read about the profile section on i-REBOUND and how to use it | # Can read my saved bookmarks section to know what the page is # Can see button to create a goal (take me to the enableme goal setting section) # Can see button to download exercise tracking sheet (pdf) |
| **Saved bookmarks media block** | As a user I want to watch a video on my profile section so I know how to use it | # Can watch a video (embeded YouTube) # Can view an image |
| **Bookmark filter** | As a user i want to filter my bookmarks | # Can read filter title  # Can filter items between [Eat well] [Moving well][Hints and Tips] |
| **Bookmark collection** | As a user i want to view my bookmarks, click on one to view it, or remove an item from my bookmarks | # Can see all items bookmarked # Can see number of items bookmarked # Can select item to read more # Can choose to remove an item from my collection |
| **Recipe page** | | |
| **Recipe intro block** | As a user i want to read an Item title and brief description, see tagged items and then have the option to save, print, share or listen to item | # Can read the title of the item # Can read a brief description of the item # Can go back to collection by clicking on back to hints and tips # Can save item to my bookmarks # Can see when i save item that it has moved to my bookmarks # Can print a print friendly version of the page # Can share item on social media (use existing 'add this' as on enableme) # Can play read speaker button to hear content read aloud # Can see icons for tagged all tagged items |
| **Recipe intro block (CMS)** | As a CMS user i can add title, description and icons and text so i can add an item to the eat well collection | # Can add title # Can add description # Can add icons and text # Can add my own button, text and link  * Control over ‘other’ button not required |
| **Recipe media block** | As a user I want to watch a video or view an image of the dish I am interested in | # Can watch a video (embeded YouTube) # Can view an image |
| **Recipe media block (CMS)** | As a CMS user I can choose to add a video or image | # Can add video link into CMS # Can add image instead of video |
| **Ingredients block** | As a user i can see what i need to undertake a recipe | # Can see the title 'What you need' # Can see for how many people these ingredients will make for # Can see individual items (text) i need to undertake a recipe |
| **Ingredients block (CMS)** | As a CMS user i can add items that you need to complete the activity | # Can add item image # Can add item text # Can add link in the text # Can choose not to have image |
| **Steps** | As a user i can see all the steps to complete a recipe so i can follow along | # Can see the title 'What to do' # Can see step by step instructions (Step#, text and image) # Can see link if required |
| **Steps (CMS)** | As a CMS user i want add step by step instructions with numbering. | # Can add multiple steps # Can add text with links in step instruction # Can add sub text item below instruction with text link # Can add image or video # Can click on image and it expands # Can click on video and it opens in lightbox. |
| **Comments block** | As a user i want to comment on a recipe | # Can add a comment to a recipe. Cannot add links, images or videos. # Can scroll down page to read more comments (no pagination) |
| **Exercise page** | | |
| **Exercise intro block** | As a user i want to read an Item title and brief description, see tagged items and then have the option to save, print, share, listen to item, or download tracking sheet | # Can read the title of the item # Can read a brief description of the item # Can go back to collection by clicking on back to hints and tips # Can save item to my bookmarks # Can see when i save item that it has moved to my bookmarks # Can print a print friendly version of the page # Can share item on social media (use existing 'add this' as on enableme) # Can play read speaker button to hear content read aloud # Can download exercise tracking sheet |
| **Exercise intro block (CMS)** | As a CMS user i can add title, description and icons and text so i can add an item to the move well collection | # Can add title # Can add description # Can add icons and text # Can add my own button, text and link  * Control over other buttons (print share, listen) not required |
| **Exercise media block** | As a user i want to watch a video or view an image of the exercise im interested in | # Can watch a video (embeded YouTube) # Can view an image |
| **Exercise media block (CMS)** | As a CMS user i can choose to add a video or image | # Can add video link into CMS # Can add image instead of video |
| **What you need block** | As a user I can see what i need to undertake an exercise | # Can see the title 'What you need' # Can see individual items (text and icon) i need to undertake an exercise |
| **What you need block (CMS)** | As a CMS user i can add items that you need to complete the activity | # Can add item image # Can add item text # Can add link in the text # Can choose not to have image |
| **instructions block** | As a user i can see all the steps to complete an exercise so i can follow along | # Can see the title 'What to do' # Can see step by step instructions (step#, text and image) # Can see link if required |
| **instructions block (CMS)** | As a CMS user i want add step by step instructions with numbering. | # Can add multiple steps # Can add text with links in step instruction # Can add sub text item below instruction with text link # Can add image or video # Can click on image and it expands # Can click on video and it opens in lightbox. |
| **Comments block** | As a user i want to comment on a recipe | # Can add a comment to a recipe. Cannot add links, images or videos. # Can scroll down page to read more comments (no pagination) |
| **Hints & Hacks page** | | |
| **H&H intro block** | As a user i want to read an Item title and brief description and then have the option to save, print or share item | # Can read the title of the item # Can read a brief description of the item # Can go back to collection by clicking on back to hints and tips # Can save item to my bookmarks # Can see when i save item that it has moved to my bookmarks # Can print a print friendly version of the page # Can share item on social media (use existing 'add this' as on enableme) # Can play read speaker button to hear content read aloud |
| **H&H intro block (CMS)** | As a CMS user i can add title, description and icons and text so i can add an item to the move well collection | # Can add title # Can add description # Can add icons and text # Can add my own button, text and link  * Control over other buttons (print share, listen) not required |
| **H&H media block** | As a user i want to watch a video or view an image of the item I’m interested in | # Can watch a video (embedded YouTube) # Can view an image |
| **H&H media block (CMS)** | As a CMS user i can choose to add a video or image | # Can add video link into CMS # Can add image instead of video |
| **H&H content block** | As a user i want to read about a hint and tip | # Can read the main content of hint and hacks |
| **H&H content block (CMS)** | As a CMS user i want to add content | Rich text |
| **Comments block** | As a user i want to comment on a recipe | # Can add a comment to a recipe. Cannot add links, images or videos. # Can scroll down page to read more comments (no pagination) |
| **Accessibility** | | |
| **Read speaker** | As a user i can play read speaker tool so i can hear back the audio of written text on a page | # Can play read speaker on pages to play back page content |
| **Mobile / tablet friendly** | As a user i want to easily use this site on mobile and tablet | # Can view the site on mobile. # Can see the site is optimised for tablet. |
