# Supplementary material for: Adapting a Telehealth Physical Activity and Diet Intervention to a Co-Designed Website for Self-Management After Stroke: Tutorial
Source: J Med Internet Res. 2024 Oct 22;26:e58419. doi: 10.2196/58419 (PMC11538875; doi:10.2196/58419)
Supplement: Multimedia Appendix 10 [file jmir_v26i1e58419_app10.docx]

### Appendix 10: CAG feedback on initial prototype.

| Website section | Specific topic | Feedback |
| --- | --- | --- |
| Landing Page | General impression  Introduction video  User feedback  “My Rebound” | - Looks easy to use, inviting.  - Introduction section is good and spells out that its co-designed.  - Colours are important. Avoid green, not a good colour for people who are visually impaired.  - Make sure the order at the top matches the banners lower down on the landing page.  - Like the idea of having an introduction video.  - Suggestion to have an intro video for each section of the website, not just the landing page.  - Add a function where a user can provide their suggestions to the website.  - Wording is confusing.  - The ability to personalise the page to your needs is good.  Suggestions:   - Need a page explaining what “My Rebound” is. - Create a “how to do” documents. - Add function to click and drag a video/content straight into the “My Rebound” page. - Create a ‘save’ button eg: “Save to my Rebound”. |
| Hints and Hacks |  | - Hints and Hacks endorsed as the name for this collection of content.  - Likely to be a well-used section. |
| Eat Well | General impressions  Font size  Recipes  Filters | - This is well set up as there is a minimum number of clicks to get to where you want to get to.  - Need to explain that the saved content goes to the “My Rebound” section.  - Font size, check that it works on iPADS/phones  - Font size looks a bit small especially on the filters.  - Print function great and it will print in printer friendly format.  - Comment section on the recipe could help with engagement which is good.  - Icons for filters not necessary on the recipe tile. (difficult to notice). If using icons, when you hover mouse over the tile it should come up better.  - Get rid of button “continue to recipe” as well as the “start cooking button”, these are confusing.  - Like the idea of breakfast/lunch dinner options.  - Get rid of the “time I have” to spend cooking section as this is different for all survivors. Prep time/cooking time is individual and is not valuable to have in the i-REBOUND website. Instead, focus on how easy the recipe is… or maybe just list the number of steps needed to make the recipe.  - Get rid of the section’s “considerations” and “dietary requirements’ … they blur into one. Could consider different colours for different sections or have one above the other if that’s possible.  - A budget recipe section suggested.  - Carefully consider working, use ‘trouble swallowing’ rather than ‘overcome swallowing difficulty’. |
| Move Well | Section title  Exercise Safety  Filters | - Change section title to “Move More” for consistency  - Consider adding a button for instructions on exercise safety.  - Build up hints about ways to make exercise safe at home.  - Need categories to go with levels of ability like sitting or one handed, be clear.  - Add an “incidental exercise” button.  Suggestions:   - Consider AI for the site to learn your preferences. - Have solid images in the filters so it is easier to see |
