# Supplementary material for: Adapting a Telehealth Physical Activity and Diet Intervention to a Co-Designed Website for Self-Management After Stroke: Tutorial
Source: J Med Internet Res. 2024 Oct 22;26:e58419. doi: 10.2196/58419 (PMC11538875; doi:10.2196/58419)
Supplement: Multimedia Appendix 11 [file jmir_v26i1e58419_app11.docx]

Appendix 11: User testing guide for survivors of stroke

| **Task** | **Instruction to User (provided by facilitator)** |
| --- | --- |
| Introduction | Today’s user testing session will help shape the development of this website.  I will ask you to complete several tasks to see how easy the site is to use.  I will ask you questions while you are completing these tasks to see your overall experience of browsing the site.  Please talk aloud as you are browsing the site and trying to find things. If you could please express and emote how you feel, both positive and negative.  If something doesn’t feel right, please tell me about it. Similarly, if something works well, please tell me why it works for you.  To help further refine the prototype we would like to record your screen during this session so that we can see how you navigate through the site. |
| Task 1:  Free solo | Please start with browsing the website to build up confidence for the tasks which will follow.  For the first few minutes I will not answer any questions but if I could please ask for you to talk out loud and share how you are feeling as you browse.     - This is to observe your initial reactions and it is important that you do this alone to mimic a situation where you are browsing a new website without support from me or anyone else. |
| First impressions | What are your initial reactions to the website?  What do you think is the purpose of the site?  You went to ‘X’ area first, is that an area of interest for you? |
| Task 2:  Find a recipe | Your friend has told you about a 'Tofu and corn' recipe that they saw on the i-REBOUND after stroke website, can you please find it?  Now that you have found it can you follow the steps of the recipe?  Do you prefer knowing how many steps or how long it will take in minutes?  What do you think of the recipe page?  What do you think of the step-by-step buttons?  Which filters do you think you would use?  How would you find some breakfast ideas? |
| Break | |
| Task 3: Find an exercise | You want to find an exercise you can do. Can you find one?  What do you think of the search filters?  How would you print out an exercise to give to a friend? |
| Task 4: Find information on healthy living/eating | You want to find information on the Mediterranean diet. Where would you look for this?  What would you expect to find in the hints and hacks section? |
| Task 5: My Rebound | You have seen a recipe that you really want to try later,  How would you save it for later? |
| Final comments | If you could change one thing or leave us with one last comment, what would it be? |
| Net promoter score | Would you use this site or recommend it to someone? on a scale 0-10 (10yes) |
| Wrap up | Next steps, option to leave further feedback via online form or email. |
