# Supplementary material for: Adapting a Telehealth Physical Activity and Diet Intervention to a Co-Designed Website for Self-Management After Stroke: Tutorial
Source: J Med Internet Res. 2024 Oct 22;26:e58419. doi: 10.2196/58419 (PMC11538875; doi:10.2196/58419)
Supplement: Multimedia Appendix 12 [file jmir_v26i1e58419_app12.docx]

Appendix 12: Proposed exercises used to develop individual exercise programs.

**IREBOUND PROGRAM**

Notes:

Seated and standing options

2.5 to 5 minutes warm up options

6—8 exercises for standing/ seated

| **WARM UP** | | |
| --- | --- | --- |
| **FUNCTION** | **EXERCISE** | **SUGGESTION** |
| ***STANDING***  ***2.5 to 5 minutes*** | 30 seconds Leg swings  60 seconds Marching on the spot  30 seconds’ lateral shuffle  30 seconds Arm circles    Repeat x 1 for 5 minute warm up | *Hold onto chair/ wall or bench for stability in leg swings & lateral shuffle.*    *Walk or jog to speed. Can be increased to faster or slower pace*    *Arm circles can be done seated if required* |
| ***SEATED***    ***2.5 to 5 minutes*** | 30 seconds Torso twists  60 seconds  Foot tap  30 seconds  Ankle circles  30 seconds knee bends to chest    Repeat x 1 for 5 minute warm up | *If only one foot is functional you can yab can tap cross your foot left to right sides of your chair with your good leg. The same can be done with knee to chest*    *Ankle circles clockwise and anticlockwise* |
|  | | |
| **PROGRAM** | | |
| **FUNCTION** | **EXERCISE** | **SUGGESTION** |
| ***STANDING*** | 30 x Heel digs with front press (single arms) – alternating sides | This can be done seated – sit towards the front of the chair    To advance this you can alternate front press and across the body press. |
| ***STANDING*** | 20-30 Squats | Hold onto the back of the chair for stability if you need.    To advance the Squat do squat jabs |
| ***STANDING*** | 30 x Side steps | Hold onto the back of the chair for stability if you need.    To advance side steps do Skaters or front/ side/ back steps |
| ***STANDING*** | 20 x each side Reverse lunge front kick    40 x if using 1 leg | Hold onto s sturdy chair or bench to stabilise your lunges    To advance this increase intensity |
| ***STANDING*** | “Ball” twists with side thrusts with body “bounce” | If you can’t hold a ball or 1.25 litre bottle with You can rotate side to side with a small bounce in between. Can be done seated    This can be done seated    To advance you can do the med ball twist with a squat between each rotation |
| ***STANDING*** | Punching-  jabs and hooks | This can be done seated if required.    If you only have use of one arm rotate your torso vigorously with each single arm punch.      To advance this increase intensity, number of jabs  Or hold a deep sumo squat |
| ***STANDING*** | Low impact jumping jacks (step and reach single arm above to the side of your head) | You can stand and reach single arm above to the side of your head. Alternate sides of head if you are using one arm.    To advance try to do a small jump |
| ***STANDING*** | Toe taps | Toe taps can be done seated.    You can stand and hold the back of a sturdy chair to make this easier.    To advance toe tabs do Step ups |
|  | | |
| ***SEATED*** | 30 x Overhead raise | You can do this with one arm.    The can increase intensity by doing this faster |
| ***SEATED*** | 30 x Ball thrusts with torso “bounce” | Push forward and in between each push bounce your torso. |
| ***SEATED*** | 30 seconds of Sitting Bicycle Crunches | This can be down with one leg – cycle with this leg left, middle to right |
| ***SEATED*** | 30 x Punching-  jabs and hooks | Increase intensity by jabbing faster and wiggling your torso |
| ***SEATED*** | 30 seconds of Aeroplane arm | Bounce your torso between rotations |
| ***SEATED*** | 30 seconds of Knee kicks | This can be down with one leg |
| ***SEATED*** | 30 x Heel digs with front press (single arms) | Sit to the front of your chair or on a couch if possible. This can be down with one arm and leg just increase the intensity |
| ***SEATED*** | 60 x Side stepping single leg    (30 x if using both legs) | Sit to the front of your chair or on a couch if possible. |
|  | | |
| **COOL DOWN** | | |
| **FUNCTION** | **EXERCISE** | **SUGGESTION** |
| ***STANDING*** | Hamstring Stretch – single leg  Lunging Calf Stretch.  Sumo Squat Stretch  Slow march | Hold onto a chair or bench for stability with the hamstring stretch and lunging calf stretch |
| ***SEATED*** | Seated calf stretch  Wrist Rolls  Overhead Shoulder Stretch  Cross body arm stretch | For wrist rolls and overhead shoulder stretch you can use your functioning limb to clasp the wrist of the affected side to achieve the range of motion    Use a towel to do the calf stretch |

**INCIDENTAL EXERCISE**

When the kettle boils do side steps or quats

Commercial break workout

Dog poo march

Walk in intervals – faster/ slower

Walking lunges between rooms

Use stairs if possible/ safe

**STRATEGIES/ TIPS**

Track exercise (Physio gave me a Fitbit to use – steps)

Chair or wall stabilisation to start

Cane as stabiliser (e.g.) lunges

Exercises in bed (e.g. leg raises)

Stretching in bed

schedule like you do an appointment

Have a plan – prepare equipment, instructions, notes etc
